# Increased levels of extracellular matrix proteins associated with extracellular vesicles from brains of aged mice

**DOI:** 10.1111/acel.14359

**Published:** 2024-10-08

**Authors:** Azariah K. Kaplelach, Charles F. Murchison, Kyoko Kojima, James A. Mobley, Andrew E. Arrant

**Affiliations:** ^1^ Center for Neurodegeneration and Experimental Therapeutics, Alzheimer's Disease Center, Evelyn F. McKnight Brain Institute, Department of Neurology University of Alabama at Birmingham Birmingham Alabama USA; ^2^ Institutional Research Core Program/Mass Spectrometry, University of Alabama at Birmingham Birmingham Alabama USA; ^3^ Department of Anesthesiology and Perioperative Medicine University of Alabama at Birmingham Birmingham Alabama USA

**Keywords:** aging, extracellular matrix, extracellular vesicles

## Abstract

Extracellular vesicles (EVs) are secreted by all major cell types of the brain, providing a mode of intercellular communication and a pathway for disposal of cellular debris. EVs help maintain healthy brain function, but may also contribute to diseases affecting the brain. EVs might contribute to aging of the brain, as aging‐related processes such as inflammation and cellular senescence may alter EV cargo, promoting further inflammation and senescence. However, the effects of aging on brain EVs and the function of EVs in the aging brain remain poorly understood. To address this question, we measured the levels and protein cargo of EVs isolated from the brains of 4‐, 12‐, and 22‐month‐old C57BL/6J mice. We detected no changes in EV levels, but observed age‐dependent changes in EV proteins. EV fractions from aged (22 month old) brains contained higher levels of extracellular matrix proteins than EV fractions from young (4 month old) brains, with intermediate levels in 12‐month‐old brains. Specifically, EV fractions from aged mice contained elevated levels of hyaluronan and proteoglycan link proteins 1 and 2 and several chondroitin sulfate proteoglycans (CSPGs). Analysis of extracellular matrix in several brain regions of aged mice revealed increased immunolabeling for the CSPG aggrecan, but reduced labeling with *Wisteria floribunda* agglutinin, which binds to chondroitin sulfate side chains of CSPGs. These data are consistent with prior studies showing changes to the composition of extracellular matrix in aged brains, and indicate a novel association of EVs with changes in the extracellular matrix of the aging brain.

## Graphical abstract

Proteomic analysis of EVs isolated from the brains of 4‐, 12‐, and 22‐month‐old mice revealed increases in chondroitin sulfate proteoglycans (CSPGs) and the link proteins Hapln1 and Hapln2. These proteins interact to form ECM in the brain, including perineuronal nets. Analysis of brains from the same mice revealed signs of altered ECM composition, indicating a potential association of altered EV cargo with age‐related changes to the brain's ECM.

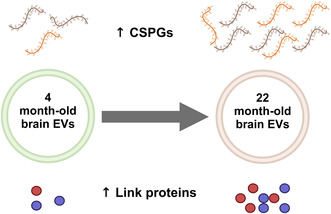

**Abbreviations**
CS: chondroitin sulfate
CSPG: chondroitin sulfate proteoglycan
ECM: extracellular matrix
EV: extracellular vesicle
FC: fold change
WFA: Wisteria floribunda agglutinin

## 1 INTRODUCTION

Extracellular vesicles (EVs) are found in most biofluids and comprise an assortment of vesicles originating from several cellular pathways. Major classes of EVs include microvesicles (approximately 100–1000 nm diameter) that bud from the cell membrane, exosomes (approximately 50–150 nm diameter) that are secreted from the endolysosomal system, and apoptotic bodies (approximately 100–5000 nm in diameter) that are generated as cells undergo apoptosis (Mathieu et al., 2019; Phillips et al., 2021; Tkach & Thery, 2016). EVs can serve as a pathway for disposal of cellular waste (Eitan et al., 2016; Mathews & Levy, 2019) or as a mode of cellular signaling by delivering proteins or RNA that modulate the function of recipient cells (Mathieu et al., 2019; Simons & Raposo, 2009; Tkach & Thery, 2016).

All major cell types in the brain secrete EVs, which perform a variety of functions (Delpech et al., 2019; Gharbi et al., 2020; Kramer‐Albers & Werner, 2023; Paolicelli et al., 2019). Under normal conditions, EVs secreted by glia promote neuronal growth, survival, and activity (Antonucci et al., 2012; Datta Chaudhuri et al., 2020; Frohlich et al., 2014; Fruhbeis et al., 2013, 2020; Wang et al., 2011; You et al., 2020). EVs secreted from neurons regulate glial function through mechanisms such as modulating astrocytic expression of glutamate transporters (Morel et al., 2013) or microglial pruning of dendritic spines (Bahrini et al., 2015). Neuronal EVs also act on endothelial cells to maintain the brain's vasculature (Dong et al., 2022; Xu et al., 2017), and on other neurons to regulate neuronal growth (Chivet et al., 2014; Liu et al., 2015). EVs can also mediate communication among glia (Willis et al., 2020) and between glia and peripheral immune cells (Dickens et al., 2017; Li et al., 2018).

Dysregulation of EV secretion or cargo may contribute to diseases affecting the brain. In cancer, EVs secreted from tumors carry proteins and microRNA that act on recipient cells to promote metastasis (Costa‐Silva et al., 2015; Fong et al., 2015; Peinado et al., 2012). These EVs can cross the blood–brain barrier and promote metastasis to the brain (Fong et al., 2015; Morad et al., 2019). EVs may also be involved in neurodegenerative disease. While EVs may provide a pathway for relieving neurons of pathologic proteins and other endolysosomal cargo (Mathews & Levy, 2019), they may also spread pathologic proteins and microRNAs throughout the brain, contributing to disease progression (Vassileff et al., 2020; You & Ikezu, 2019).

There is growing evidence that aging alters the levels and cargo of EVs throughout the body. Aging‐related processes such as inflammation, cellular senescence, and impaired autophagy (Lopez‐Otin et al., 2023; Mattson & Arumugam, 2018) may increase EV secretion (Eitan et al., 2016; Mathews & Levy, 2019; Takasugi, 2018), and are associated with changes to EV cargo (Basisty et al., 2020; Delpech et al., 2019; Takasugi, 2018; Yang et al., 2018). Aging appears to affect EV secretion in the brain. Brains from aged female mice exhibit increases in EVs relative to young adult female mice, and brains from aged male and female mice exhibit increases in mitovesicles (Kim et al., 2022), an EV subtype secreted in relation to mitophagy (D'Acunzo et al., 2021). EV secretion also increases over time during long‐term culture of primary neurons (Guix et al., 2021). The cargo of brain EVs might also change with age, as EVs from the brains of aged cynomolgus monkeys contain higher levels of amyloid β than EVs from younger monkeys (Koinuma et al., 2021).

EVs from aged people and animals exhibit functional changes that may further drive aging‐related processes such as inflammation and senescence (Yin et al., 2021). EVs from aged human (Eitan et al., 2017) and mouse (Alibhai et al., 2020) plasma promote inflammation more than EVs from young plasma. EVs secreted from senescent cells are capable of inducing senescence‐related phenotypes in nearby cells (Takasugi, 2018; Wallis et al., 2021). EVs may also lose beneficial effects with age, as treating aged mice with EVs from young mouse plasma exerts beneficial effects throughout the body (Chen et al., 2024). Similar changes may occur in humans, as EVs from aged human plasma contain less mitochondrial DNA and are less able to stimulate cellular respiration than EVs from young human plasma (Lazo et al., 2021).

Though there is evidence that EV levels and cargo change with age and may contribute to aging‐related dysfunction, the role of EVs in aging of the brain is poorly understood. In an effort to better understand the potential role of EVs in the aging brain, we analyzed the levels and protein cargo of brain EVs from young (4 month old), middle‐aged (12 month old), and aged (22 month old) C57BL/6J mice. Here, we report similar levels of EVs across age groups, but changes to the protein cargo of aged EVs that may be associated with aging‐related changes in the brain's extracellular matrix.

## 2 MATERIALS AND METHODS

### 2.1 Animals

Male and female C57BL6/J mice (#000664) were ordered from the Jackson Laboratory at ages 3, 11, or 21 months and kept in a pathogen‐free barrier facility accredited by the Association for Assessment and Accreditation of Laboratory Animal Care International. Mice were kept for 1 month prior to brain collection, so that they were aged 4, 12, or 22 months at the end of the study. Mice were maintained on a 12 h light: dark cycle, with lights on at 6 AM and off at 6 PM, and given free access to food (Envigo #7917) and water. Aside from routine inspections and weekly bedding changes, mice were undisturbed prior to brain collection.

For brain collection, mice were anesthetized with 200 mg/kg pentobarbital (Euthasol, Virbac), then transcardially perfused with 0.9% saline. Brains were removed and bisected into hemibrains, which were either frozen on dry ice for EV isolation or fixed for 48 h at 4°C in 4% paraformaldehyde (MilliporeSigma).

### 2.2 Brain EV isolation

Brain EVs were isolated from frozen hemibrains as previously described (Arrant et al., 2020) using a method adapted from Vella et al., 2017. Hemibrains were weighed, sliced into 1–2 mm slices, then digested in collagenase type 3 (Worthington) diluted in Hibernate A medium (ThermoFisher) at 60 units of enzyme per 100 mg tissue. Samples were digested for 15 min at 37°C on an orbital shaker at 225 rpm, triturated three times with a 10 mL serological pipette, and then digested for another 5 min before addition of protease inhibitors (Halt protease inhibitor cocktail, ThermoFisher) and trituration with a 5 mL serological pipette.

A small volume of the resulting digest was collected as a control sample (referred to as “digest” in the manuscript). The rest of the digest was subjected to differential centrifugation to remove cells and cellular debris (successive centrifugation at 300, 2000, and 10,000 × *g*). The final supernatant was then spun over a discontinuous sucrose gradient (0.6, 1.3, and 2.5 M sucrose) at 180,000 × *g* for 3 h using a Beckman Coulter SW 41 Ti rotor. After removing the upper layer of PBS, four fractions were collected, diluted in PBS, and pelleted at 11,015,000 × *g* for 70 min using a Beckman Coulter 50.4 Ti rotor. Pellets were resuspended in 40 μL of PBS.

### 2.3 Iodixanol gradient centrifugation

EVs isolated as described above were diluted in PBS and loaded over a discontinuous iodixanol (MilliporeSigma) density gradient, ranging from 5% to 40% iodixanol. Gradients were centrifuged for 16 h at 100000 × *g* using a Beckman Coulter SW 41 Ti rotor and harvested in 1 mL fractions. An aliquot of each fraction was collected to determine iodixanol density by measuring absorbance at 340 nm. Fractions were further diluted with PBS and pelleted at 11,015,000 × *g* for 70 min using a Beckman Coulter 50.4 Ti rotor for analysis by immunoblot.

### 2.4 Nanoparticle tracking analysis

EV samples were diluted 1:500 in PBS prior to analysis on a Nanosight NS300 imaging system (Malvern Panalytical). Total particle concentration and concentration binned by particle size (10 nm increments) were used to compare age groups.

### 2.5 Immunoblotting

Markers for EVs, other organelles, and extracellular matrix proteins were analyzed by immunoblot of EV fractions and brain digests. EV fractions were analyzed with volumetric immunoblotting by diluting 12.5 μL of each fraction with 12.5 μL Laemmli buffer (120 mM Tris, 4% SDS, 20% glycerol, 0.02% bromophenol blue, pH 6.8), heating for 5 min at 95°C, and loading 20 μL onto 10% TGX polyacrylamide gels (Bio‐Rad). The resulting data from EV fractions were normalized to brain weight to account for differences in the amount of source tissue. Two technical replicates of the blots for EV marker proteins were averaged to provide the results shown in Figure 2.

Brain tissue digests and pellets had protein content analyzed by BCA assay (ThermoFisher) and were diluted to uniform protein concentration before dilution with Laemmli buffer, heating, and loading onto polyacrylamide gels as described above.

After SDS‐PAGE, proteins were transferred to Immobilon‐FL PVDF membranes (MilliporeSigma). Membranes were blocked with protein‐free blocking solution (ThermoFisher) prior to overnight incubation with primary antibodies. Membranes were then incubated with species‐matched IRDye‐conjugated secondary antibodies (Li‐COR Biosciences) and imaged on an Odyssey scanner (Li‐COR Biosciences).

The following primary antibodies were used for immunoblots: CD81 (sc‐166029, mouse monoclonal, Santa Cruz Biotechnology), Flotillin‐1 (610820, mouse monoclonal, BD Transduction Laboratories), HSP‐70 (sc‐32239, mouse monoclonal, Santa Cruz Biotechnology), Grp94 (sc‐32249, rat monoclonal, Santa Cruz Biotechnology), Cytochrome C (sc‐13156, mouse monoclonal, Santa Cruz Biotechnology), Histone H3 (4499, rabbit monoclonal, Cell Signaling Technology), Hapln1 (AF2608, goat polyclonal, R&D Systems), and Hapln2 (sc‐376797, mouse monoclonal, Santa Cruz Biotechnology and NBP1‐91977, rabbit polyclonal, Novus Biologicals).

### 2.6 Electron microscopy

For basic visualization of EVs, EV fractions were diluted 1:5 in TBS and pipetted onto copper grids. Grids were briefly stained with 2% uranyl acetate before imaging. For immuno‐electron microscopy, EV preparations were diluted 1:10 in PBS with 0.1% BSA and incubated overnight with an anti‐Neurocan antibody (AF5800, sheep polyclonal, R&D systems) or with no primary antibody as a control. The EV/antibody solution was then fixed in a final concentration of 2% paraformaldehyde and pipetted onto formvar/carbon coated nickel grids (Electron Microscopy Sciences). Grids were washed, then incubated with a donkey anti‐goat secondary antibody conjugated to 10 nm nanogold particles (#25551–21, Electron Microscopy Sciences). Grids were washed, fixed in 2% glutaraldehyde, and then stained with 2% uranyl acetate prior to imaging. All images were obtained with a JEOL JEM 1400 electron microscope.

### 2.7 Immunostaining

After collection and fixation as described above, mouse hemibrains were cryoprotected in 30% sucrose and sliced into 30 μm sections on a sliding microtome. Fluorescent aggrecan immunostaining was conducted as previously described (Palop et al., 2011). Brain sections were incubated overnight with anti‐aggrecan primary antibody (AB1031, rabbit polyclonal, MilliporeSigma) and fluorescein‐conjugated *Wisteria floribunda* Agglutinin (Vector Laboratories) in 3% bovine serum albumin (Fisher Scientific). The following day, sections were labeled with Alexafluor 594‐conjugated goat anti‐rabbit secondary antibody (ThermoFisher), then stained with 1% Sudan Black B (Electron Microscopy Sciences) to quench age‐related autofluorescence. Sections were mounted onto Colorfrost Plus slides (Fisher Scientific) and coverslipped with Prolong Gold (ThermoFisher) prior to imaging.

Slides were coded by mouse number and imaged at 10X using an EVOS M5000 microscope (ThermoFisher) using uniform lamp settings at which pixel intensities were not saturated. Three images were taken of each brain region from each mouse, and data from these images were averaged to provide a single data point per mouse. Fluorescent intensity was measured using ImageJ (Schindelin et al., 2015) by applying a uniform lower threshold to exclude background fluorescence, then measuring the average intensity of all pixels above that threshold.

For chromogenic immunostaining, sections were incubated with anti‐aggrecan primary antibody (AB1031, rabbit polyclonal, MilliporeSigma) as described above, followed by biotinylated anti‐rabbit antibody (Vector Laboratories), and VectaStain Elite avidin‐biotin complex reagent (Vector Laboratories). Labeling was detected with diaminobenzidine (MP Biomedicals). Sections were then mounted onto Colorfrost Plus slides (Fisher Scientific), cleared with xylene, and coverslipped with Permaslip (Alban Scientific). These slides were imaged with an EVOS M5000 microscope at 10X using a uniform lamp setting. Three images were taken of each region per mouse and results were averaged to provide a final value for each mouse. Images were quantified using ImageJ by applying a uniform threshold and measuring the percent of total image area covered by pixels darker than the threshold value.

### 2.8 Mass spectrometry

EV protein contents were analyzed by label‐free mass spectrometry as previously described (Arrant et al., 2020; Ludwig et al., 2016). Briefly, 5 μg of protein from each EV sample was loaded onto 10% Bis‐Tris gels and subjected to SDS‐PAGE. Each lane was cut into three fractions and digested overnight with mass spectrometry grade trypsin (Promega). Peptides were analyzed by LC–MS on a ThermoFisher Q Extractive HF‐X system. Data were searched on MASCOT (Matrix Science) and filtered using Scaffold (Proteome Software). Relative quantification was performed by normalized spectral counting (Liu et al., 2004; Old et al., 2005).

### 2.9 Experimental design and statistics

Comparison of EV levels or extracellular matrix proteins between age groups was performed by one‐way ANOVA. EV concentration binned by size was analyzed by two‐way repeated measures ANOVA with factors of age and size. Aggrecan and WFA labeling were analyzed by two‐way repeated measures ANOVA with factors of age and brain region. Significant main effects or interactions were followed by post hoc testing as described in the figure legends. All data were analyzed to test assumptions of normal distribution by Shapiro–Wilk test and of equal variance by Brown–Forsythe test and Bartlett's test. Data not meeting these assumptions were log transformed prior to analysis, with the exception of levels of aggrecan and versican detected by mass spectrometry, which included many zero values in 4‐month‐old mice and were thus analyzed by Kruskal–Wallis test. Two‐tailed tests were used for all analyses, with α set at 0.05. These analyses were conducted with GraphPad Prism 10.

Gene ontology analysis was performed using the clusterprofiler package in R (G. Yu et al., 2012), with *p* values adjusted for FDR using the Benjamini‐Hochberg method. Prior to pair‐wise comparison of each age group, we excluded any proteins that were not detectable in at least four samples per age group. We then performed principal components analysis and visualized the results using the ggfortify package in R (Tang, 2016). No mice were excluded from further analysis. Pairwise differential protein analysis was performed using the R Bioconductor package ROTS (Reproducibility‐optimized Test Statistic) for all pairings of age groups (Suomi et al., 2017) using the following settings, *B* = 1000, *K* = 900, seed = 1. Proteins with differential abundance between 4‐ and 22‐month‐old mice at FDR <0.1 were subjected to gene ontology analysis using the clusterprofiler package (G. Yu et al., 2012), with *p* values adjusted for FDR using the Benjamini‐Hochberg method.

## 3 RESULTS

### 3.1 Similar levels of brain EVs in 4‐, 12‐, and 22‐month‐old mice

**FIGURE 1:**
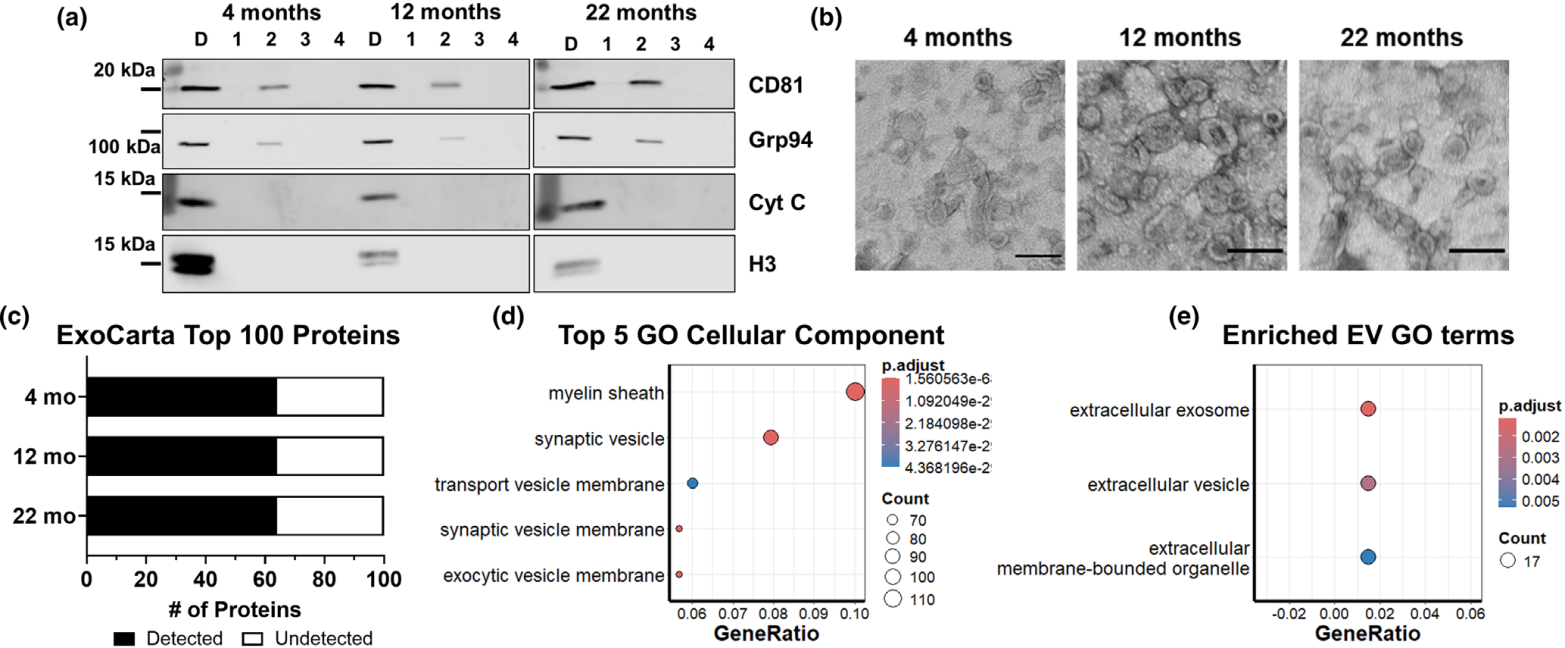
Characterization of Brain EVs. EVs were isolated from hemibrains of mice aged 4, 12, or 22 months using a previously described protocol involving gentle tissue dissociation, differential centrifugation, and ultracentrifugation over a sucrose gradient (Arrant et al., 2020; Vella et al., 2017). (a) The sucrose gradient yielded four fractions, with EV markers enriched in the second fraction, which was used for all subsequent analyses. The EV fraction was consistently negative for markers of nuclei (H3 = histone H3) and mitochondria (Cyt C = cytochrome C), but contained low levels of the endoplasmic reticulum marker Grp94. (b) However, electron microscopy revealed that the EV fraction was highly enriched for vesicles with typical EV size and morphology (scale bars = 100 nm). (c) EV enrichment was further confirmed by mass spectrometry (*n* = 6 mice per age group), which detected 64 of the top 100 EV proteins defined by the Exocarta database (Keerthikumar et al., 2016) in each age group. (d) Gene ontology analysis of the proteins detected among all age groups revealed enrichment of several terms associated with vesicular membranes (Top 5 significant GO Cellular Component terms shown in d), as well as GO Cellular Component terms associated with EVs (e).

We isolated EVs using a protocol previously adapted for frozen mouse brain tissue (Arrant et al., 2020; Vella et al., 2017). This protocol yields four fractions, with EV markers concentrated in the second fraction (Figure 1a). Though negative for other organelle markers, the EV‐containing fraction contained low levels of Grp94, which is typically located in the endoplasmic reticulum. This may indicate the presence of endoplasmic reticulum proteins in the EV fractions, an issue that has been noted in other studies of EVs isolated from mouse brain tissue (Polanco et al., 2016; Vassileff et al., 2024). However, Grp94 has also been detected in EVs from several types of cultured cells (Keerthikumar et al., 2016). Electron microscopy revealed that EV fractions primarily contained vesicles ranging from 100 to 200 nm in diameter, with typical EV morphology (Figure 1b).

We also used proteomic analysis of EV fractions from each age group to determine levels of EV markers. EV fractions from each age group contained 64 of the top 100 EV proteins from the ExoCarta database (Figure 1c) (Keerthikumar et al., 2016). Gene ontology analysis of each age group revealed nearly identical results, so the groups were combined for a single analysis that revealed enrichment of GO terms for general vesicular markers (Figure 1d) and GO terms related to EVs (Figure 1e). Thus, brain EV fractions isolated from each age group were enriched for vesicles exhibiting the typical size, morphology, and protein markers of EVs.

**FIGURE 2:**
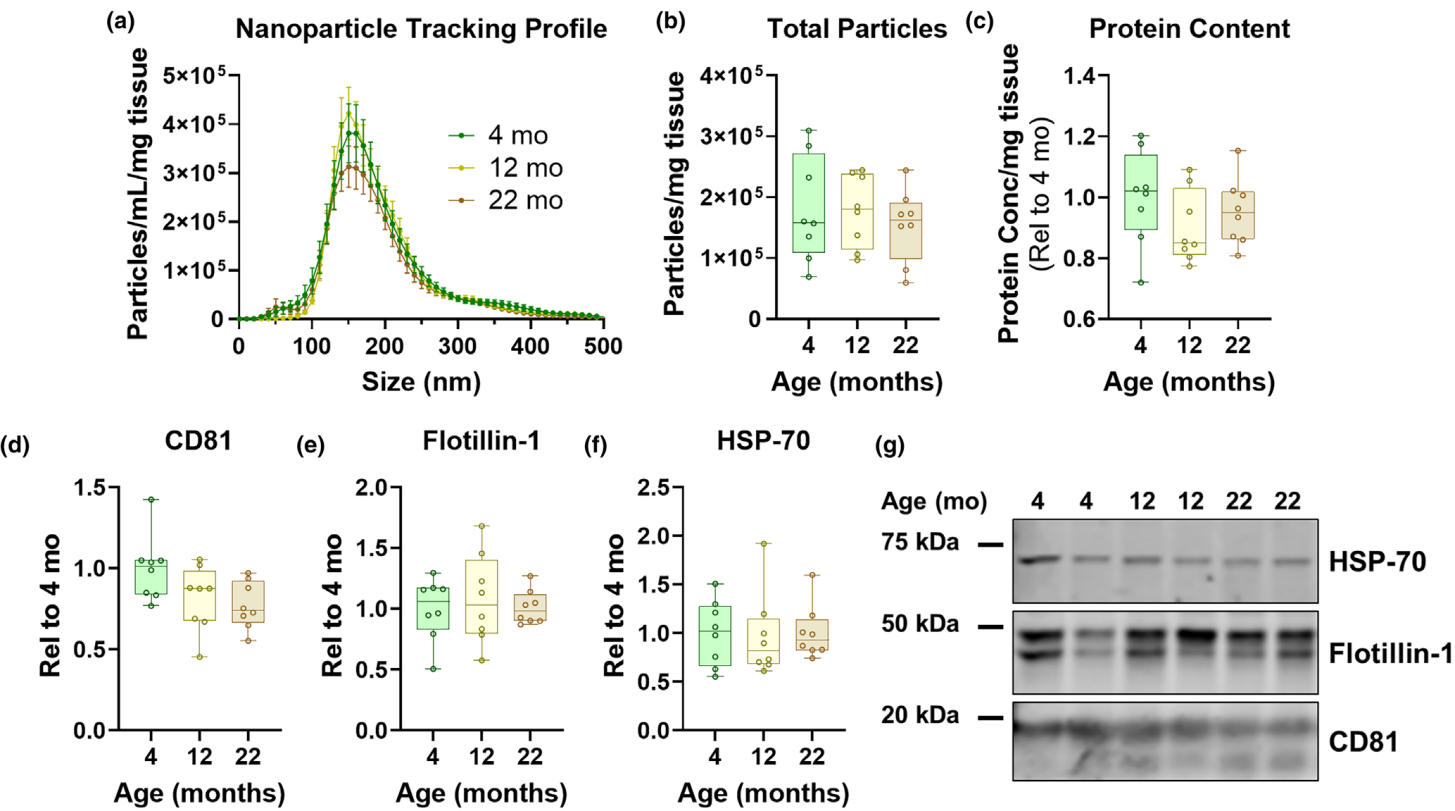
No changes in brain EV levels in aged mice. (a,b) Nanoparticle tracking analysis of the EV fraction revealed similar particle concentration and size among mice of all three age groups (a, RM ANOVA, effect of age, *p* = 0.7003, size x age, *p* = 0.8989, b, ANOVA, *p* = 0.7026). (c) Analysis of EV protein levels by BCA assay revealed similar total protein content in EVs from each age group (ANOVA, *p* = 0.3267). (d–g) Immunoblot for EV markers also revealed no significant age differences (d, CD81, ANOVA, *p* = 0.0509, e, Flotillin‐1, ANOVA, *p* = 0.8225, f, HSP‐70, ANOVA, *p* = 0.8886). EV markers were analyzed by volumetric immunoblot to assess the relative levels of EVs recovered from each mouse. Particle concentration and immunoblot data were normalized to the wet weight of the hemibrain used for EV isolation to correct for any differences in the amount of tissue used for EV isolation. *n* = 8 mice per age group (four males and four females).

Nanoparticle tracking analysis of EV fractions from brains of each age group revealed similar concentrations and sizes of particles (Figure 2a,b). Similarly, analysis of total protein content (Figure 2c) and levels of the EV markers CD81 (Figure 2d), flotillin‐1 (Figure 2e), and HSP‐70 (Figure 2f) revealed no statistically significant differences between age groups.

A recent study reported sex differences in brain EV markers in mice (Kim et al., 2022), so we analyzed EV markers by sex. We detected no significant age‐related changes in brain EVs in either male or female mice (Figure S1). However, splitting the data by sex produced a small sample size per group (*n* = 4 per age per sex), resulting in low statistical power to detect sex differences in EV levels. Additional reasons for the lack of sex differences could be the use of a different EV isolation protocol or measurement of different EV markers.

### 3.2 Proteomic analysis reveals elevated extracellular matrix proteins in brain EV fractions from aged mice

**FIGURE 3:**
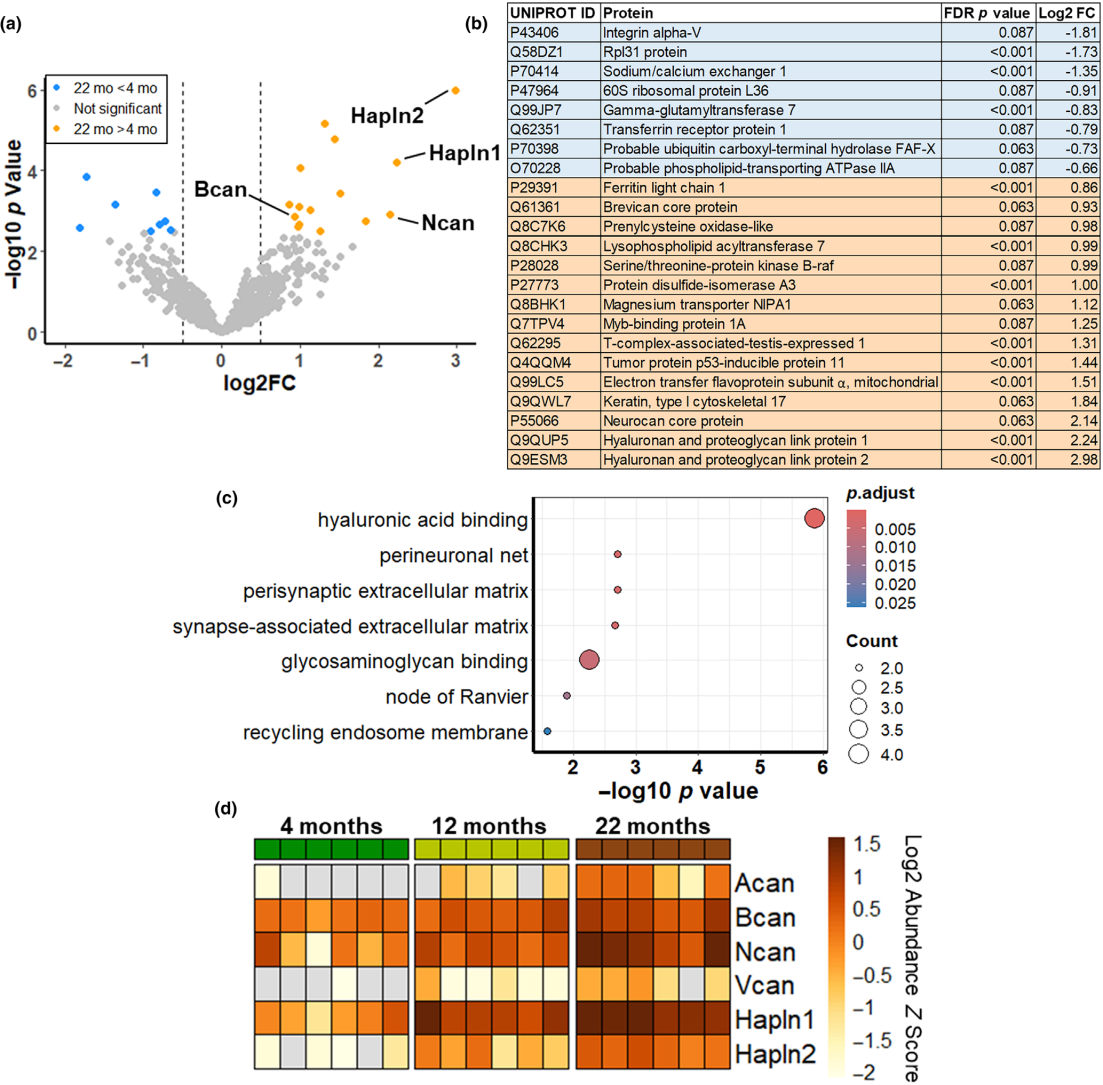
Increased ECM proteins in brain EVs from 22‐month‐old mice. a, Pair‐wise comparison of proteins detected in EVs revealed 23 proteins with differential abundance between 4‐ and 22‐month‐old mice (FDR‐corrected *p* value <0.1). (b) Summary of proteins with differential abundance from pair‐wise comparisons in (a). Proteins increased in 22‐month‐old mice are shaded orange and proteins decreased in 22‐month‐old mice are shaded blue. (c) Gene ontology analysis of these proteins primarily detected terms associated with ECM. These terms were driven by the CSPGs brevican (Bcan) and neurocan (Ncan), as well as hyaluronan and proteoglycan link proteins 1 and 2 (Hapln1 and Hapln2). (d) Comparison of the abundance of these proteins, as well as two other CSPGs that were excluded from pair‐wise analysis due to low abundance in 4‐month‐old mice (aggrecan = Acan & versican = Vcan), revealed progressive increases in each protein with age. *n* = 6 mice per age group (three males and three females). Vertical dashed lines in (a) represent a log2 fold change of ±0.5. Data in (d) are shown as *Z* scores calculated from the log2 abundance values of Acan, Bcan, Ncan, Vcan, Hapln1, and Hapln2.

We next compared the protein contents of the brain EV fraction from 4‐, 12‐, and 22‐month‐old mice using mass spectrometry. Quantitative values of all detected proteins are provided in Table S1. Principal components analysis revealed that EVs from 12‐ and 22‐month‐old mice clustered distinctly from EVs from 4‐month‐old mice (Figure S2). To detect changes in specific proteins with aging, we performed pair‐wise comparisons of protein abundance between each age group, with a threshold of FDR‐corrected *p* value <0.1 and log2 fold change of ±0.5. After correcting for multiple comparisons, we detected very few differences between 4‐ and 12‐month‐old mice or between 12‐ and 22‐month‐old mice (Figure S2). However, 23 proteins exhibited differential abundance between 4‐ and 22‐month‐old mice (Figure 3a,b).

Gene ontology analysis of these 23 differentially‐abundant proteins revealed enrichment of terms related to the brain's extracellular matrix (ECM) (Figure 3c). These results were driven by hyaluronan and proteoglycan link proteins 1 and 2 (Hapln1 and 2) and the chondroitin sulfate proteoglycans (CSPGs) neurocan and brevican, all of which were elevated in EVs from 22‐month‐old mice (Figure 3a,b). Hapln1 and 2 bind to CSPGs and connect them to hyaluronan in the brain's extracellular matrix (Fawcett et al., 2019). These interactions are particularly important for the structure of perineuronal nets (PNN), a form of ECM encasing primarily parvalbumin‐positive interneurons throughout the brain (Hartig et al., 1992; Lupori et al., 2023).

In addition to neurocan and brevican, our proteomic analysis detected the CSPGs aggrecan and versican. However, these proteins were excluded from our global pair‐wise analysis because they were undetectable in all but one of the 4‐month‐old mice (Figure 3d). Statistical analysis of these two CSPGs also revealed increases in 22‐ versus 4‐month‐old mice (aggrecan, Kruskal–Wallis test, *p* = 0.0009, versican, Kruskal–Wallis test, *p* = 0.0017), but not 12‐ versus 4‐month‐old mice.

In summary, our proteomic analysis of mouse brain EVs revealed a progressive age‐related increase in ECM proteins involved in formation of PNNs, particularly the link proteins Hapln1 and Hapln2 and the CSPGs brevican, neurocan, aggrecan, and versican (Figure 3d).

### 3.3 Confirmation of ECM proteins in aged brain EV fractions

**FIGURE 4:**
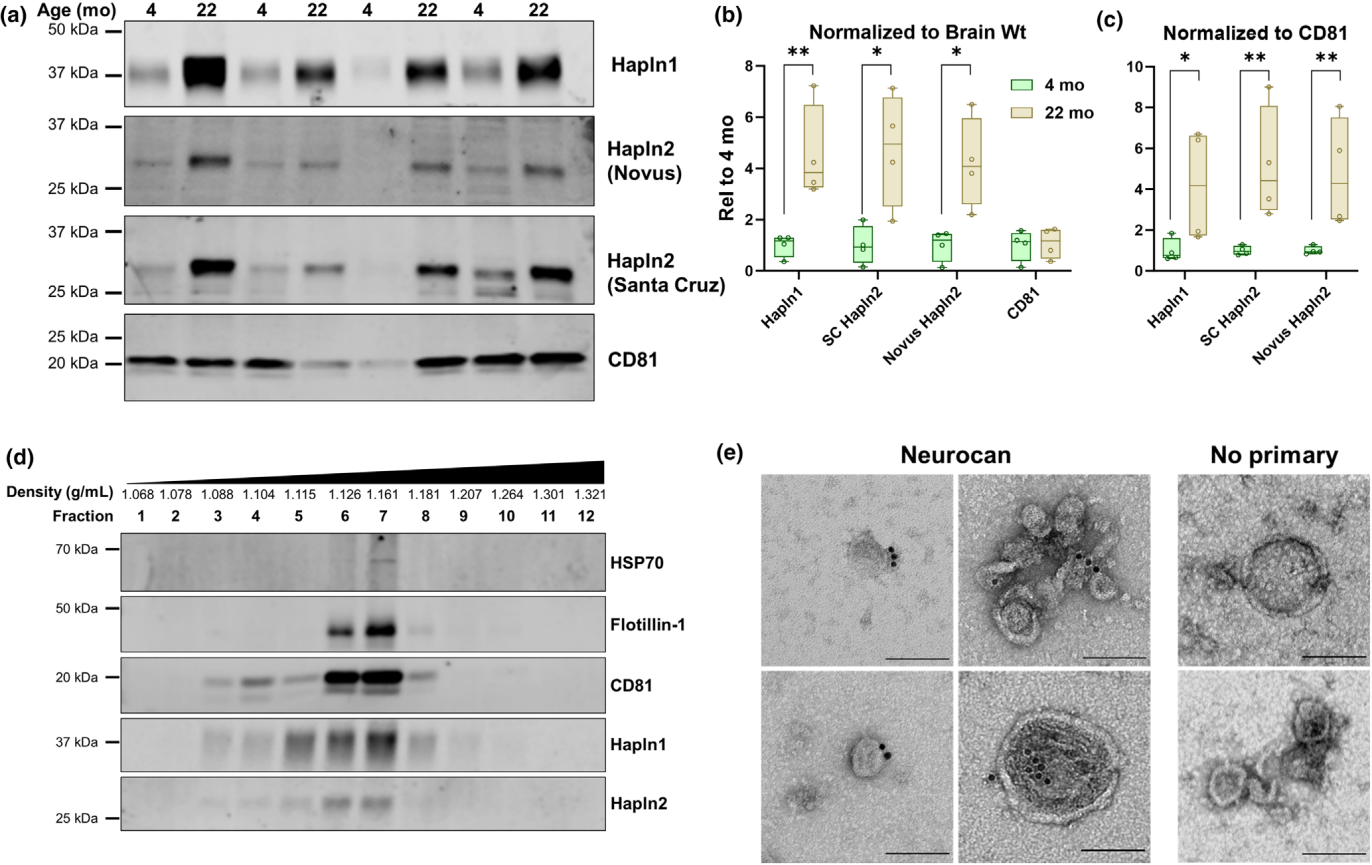
Confirmation of Hapln1 and Hapln2 in brain EVs. (a) To validate the findings from mass spectrometry analysis, EVs were isolated from a new group of 4‐ and 22‐month‐old mice (*n* = 4 per age, two males and two females). (b) Levels of Hapln1 (*t* test, *p* = 0.0045) and Hapln2 (Santa Cruz antibody, *t* test, *p* = 0.0269, Novus antibody, *t* test, *p* = 0.0313) were increased in 22‐month‐old mice when normalized to brain weight, while levels of the EV marker CD81 did not differ between ages (*t* test, *p* = 0.7337). (c) One mouse per age had low CD81 levels, so Hapln1 (*t* test, *p* = 0.025) and Hapln2 (Santa Cruz antibody, *t* test, *p* = 0.0014, Novus antibody, *t* test, *p* = 0.0032) were normalized to CD81, yielding similar results. *n* = 4 mice per age group, two males and two females. (d) Brain EVs from 22‐month‐old mice were centrifuged over an iodixanol gradient, and the resulting fractions were analyzed for Hapln1, Hapln2, and the EV markers CD81, Flotillin‐1, and HSP‐70. Both Hapln1 and Hapln2 were located in similar fractions as the EV markers, suggesting an association of these proteins with brain EVs. (e) Immunogold staining revealed neurocan immunoreactivity on EVs isolated from the brains of aged mice, confirming the presence of CSPGs on aged EVs. Scale bars represent 100 nm.

We attempted to replicate our proteomic findings with immunoblot of EVs from a new cohort of 4‐ and 22‐month‐old mice. Due to the limited amount of sample and the need to treat samples with chondroitinase to separate CSPGs by SDS‐PAGE, we limited these immunoblots to the link proteins Hapln1 and 2. The Hapln1 antibody used for this analysis has been previously used for mouse brain (Sugitani et al., 2021). We also used two Hapln2 antibodies, both of which detected the same band in EV fractions (Figure 4a). These immunoblots confirmed elevation of Hapln1 and 2 in EVs from 22‐month‐old mice (Figure 4a–c). To further validate the presence of Hapln1 and 2 in or on EVs, we centrifuged EVs over an iodixanol gradient and confirmed similar migration of Hapln1 and 2 with the EV markers CD81, Flotillin‐1, and HSP‐70 (Figure 4d). This analysis was repeated on four independent EV preparations.

To determine if the elevation of Hapln1 and 2 in aged EVs might be associated with a general increase of Hapln1 and 2 in brain tissue, we immunoblotted the crude brain digest used for EV isolation (Figure S3A), as well as the P1 pellet from the first centrifugation which should contain intact cells and insoluble material from the brain digestion. Levels of Hapln1 were increased in the P1 pellet (Figure S3D,E), consistent with prior studies of aged rodent brain tissue (Raghunathan et al., 2018; Vegh, Rausell, et al., 2014). However, despite their similar findings in brain EV fractions, the two Hapln2 antibodies produced different results. The Santa Cruz antibody revealed a large increase in Hapln2 in both the crude digest and P1 pellet (Figure S3B–E), with very low Hapln2 levels in 4‐month‐old mice. However, the Novus antibody detected faint bands in all samples, with no significant age difference. Given results from prior studies (Raghunathan et al., 2018; Vegh, Rausell, et al., 2014), these data indicate that the elevation of Hapln1 and 2 in aged EVs are likely to be associated with a general elevation of these proteins in brain tissue of aged mice.

To confirm the presence of CSPGs in aged EV fractions, we performed immuno‐electron microscopy for neurocan, the CSPG with the greatest increase in aged EVs from our global proteomic analysis (Figure 3a,d). We observed immunogold labeling of neurocan on EVs from three independent preparations from 22‐month‐old mice (Figure 4e). These immunogold particles typically seemed to be associated with the surface of EVs.

### 3.4 Altered composition of extracellular matrix in aged mouse brain

**FIGURE 5:**
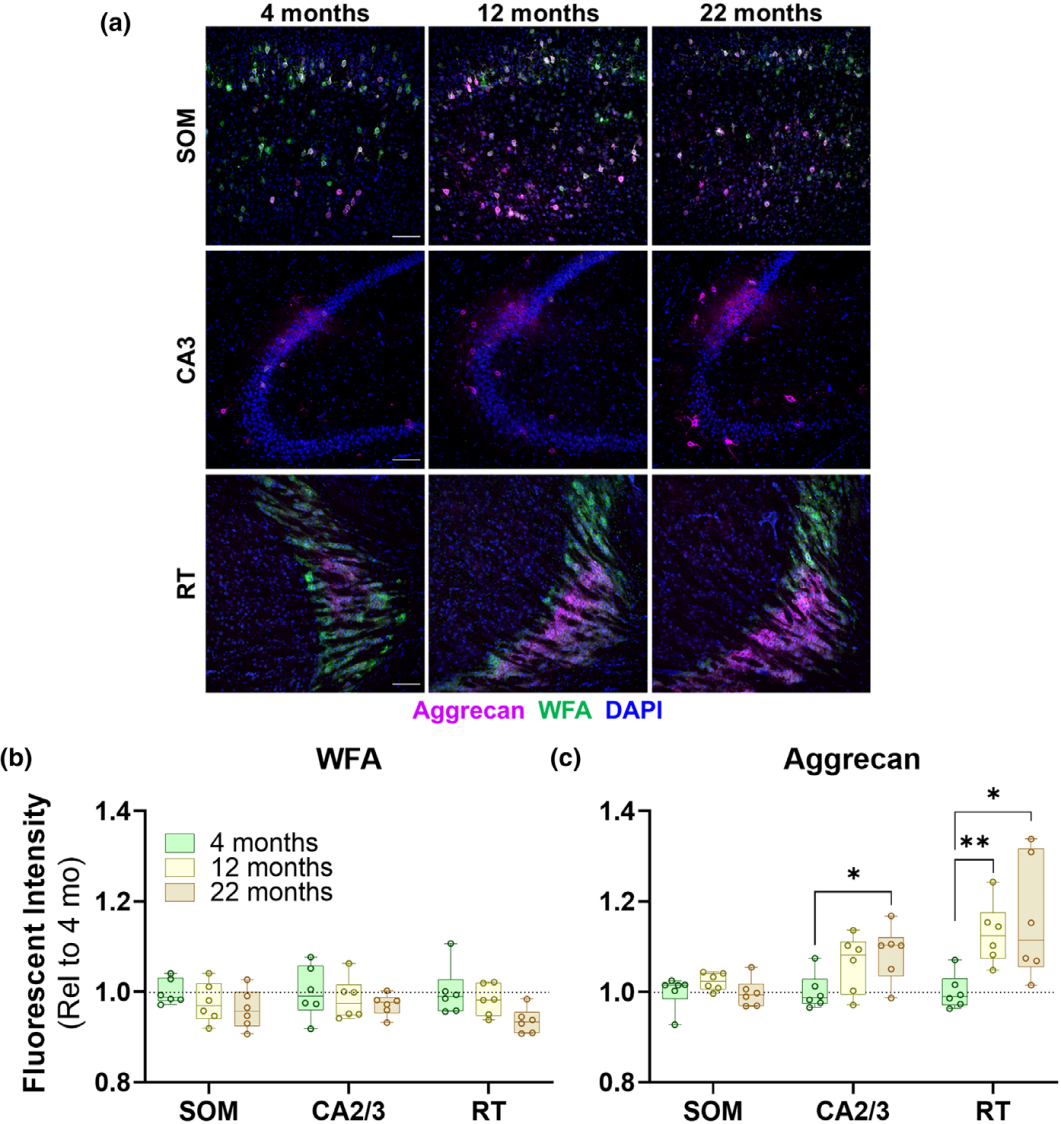
Altered ECM labeling in aged mouse brain. (a) We analyzed ECM in brains of 4‐, 12‐, and 22‐month‐old mice using *Wisteria floribunda* agglutinin (WFA) labeling and aggrecan immunostaining of somatosensory cortex (SOM), CA2/3 of hippocampus, and the reticular nucleus of the thalamus (RT). (b) Aged mice had a general reduction in the intensity of WFA labeling (RM ANOVA effect of age, *p* = 0.0247), though no individual region reached significance in post hoc testing. (c) In contrast, aged mice exhibited increased intensity of aggrecan immunolabeling in CA2/3 and RT relative to young mice (RM ANOVA effect of age, *p* = 0.0032, age x brain region, *p* = 0.0474). * = *p* < 0.05, ** = *p* < 0.01 by Dunnett's post hoc test, *n* = 6 mice per age, three males and three females. Scale bars in (a) represent 100 μm.

Since the proteins that were elevated in aged mouse brain EV fractions are key components of PNNs, we next investigated whether aged mice exhibited changes in PNNs or other forms of ECM. PNNs may be detected by several methods, including labeling with *Wisteria floribunda* agglutinin (WFA), which binds to N‐acetylgalactosamine on the chondroitin sulfate side chains of CSPGs (Hartig et al., 1992) or by immunostaining for aggrecan, the core CSPG necessary for PNN formation (Giamanco et al., 2010; Matthews et al., 2002; Rowlands et al., 2018). Prior studies in rodents report increases in PNN number or size when measuring aggrecan immunolabeling (Ferreira et al., 2023; Tanaka & Mizoguchi, 2009), but show more variable, brain region‐dependent changes when measuring WFA labeling (Brewton et al., 2016; Cisneros‐Franco et al., 2018; Mafi et al., 2020; Reed et al., 2018; Richard et al., 2018; Vegh, Heldring, et al., 2014; Vegh, Rausell, et al., 2014). We therefore employed both methods to measure PNNs in the remaining hemibrain from the mice used for proteomic analysis of brain EVs (Figure 5a). Aging‐related autofluorescence was quenched with Sudan Black B.

We analyzed several brain regions, including the somatosensory cortex, which contains many PNNs around parvalbumin‐positive interneurons (Lupori et al., 2023), CA2 and CA3 of the hippocampus, which are robustly immunolabeled for aggrecan (Dauth et al., 2016), and the reticular nucleus of the thalamus (RT), which contains many parvalbumin‐positive neurons and is thus heavily labeled by both WFA and aggrecan (Dauth et al., 2016; Lupori et al., 2023).

Analysis of WFA and aggrecan revealed divergent results. WFA labeling exhibited a general decrease in intensity with age across all brain regions (Figure 5b, RM ANOVA effect of age, *p* = 0.0247), though this did not reach significance in any individual region after correction for multiple comparisons. In contrast, aggrecan immunolabeling was more intense in both CA2/3 and RT of 22‐month‐old mice (Figure 5c), with no change in intensity in the somatosensory cortex. The increase in aggrecan immunolabeling with age was confirmed by chromogenic immunostaining for aggrecan (Figure S4), which revealed age‐related increases in the total area of aggrecan immunolabeling in somatosensory cortex and RT.

These data suggest changes to composition of the brain's ECM with age. The increased aggrecan immunolabeling indicates increased deposition of the core CSPG of PNNs, but the decrease in WFA labeling indicates potentially reduced density of chondroitin sulfate side chains of CSPGs with age. Based on our data and prior studies (Brewton et al., 2016; Cisneros‐Franco et al., 2018; Ferreira et al., 2023; Mafi et al., 2020; Reed et al., 2018; Richard et al., 2018; Tanaka & Mizoguchi, 2009; Vegh, Heldring, et al., 2014), these changes may vary by brain region.

## 4 DISCUSSION

In this study, we analyzed the levels and protein contents of EV fractions from brains of young adult (4 month old), middle‐aged (12 month old), and aged (22 month old) C57Bl/6J mice. We did not detect significant changes in EV levels with age, but found a progressive age‐related increase in the levels of ECM proteins in EV fractions. While these changes reached statistical significance only between 4‐ and 22‐month‐old mice, 12‐month‐old mice exhibited intermediate levels of ECM proteins (Figure 3d), suggesting that levels of ECM proteins in EVs may begin increasing sometime before 12 months of age. We also observed changes in ECM composition in aged brains, with increases in aggrecan immunolabeling, but decreases in chondroitin sulfate (CS) labeling with WFA. These data suggest a novel association of changes in EV cargo with changes in ECM of the aging brain, and raise several questions for future investigation.

Prior studies of ECM and PNNs in the aging brain report changes in levels of ECM structural proteins and the density and composition of CS side chains. These changes may contribute to cognitive aging, as well as aging‐related diseases of the brain. As described below, our findings of increased aggrecan immunolabeling, but decreased WFA labeling are consistent with results from these prior studies.

Levels of ECM proteins generally increase with aging in the rodent brain. Aged rodents have more aggrecan‐positive cells in prefrontal cortex and hippocampus (Tanaka & Mizoguchi, 2009) and more aggrecan‐positive puncta in the dentate gyrus (Ferreira et al., 2023) than young rodents. Immunoreactivity for hyaluronic acid‐binding protein increases in the cortex and cerebellum of aged mice (Reed et al., 2018). Synaptosomal preparations from aged mice have increased levels of Hapln1, brevican, and neurocan, indicating a potential increase in ECM around synapses with age (Vegh, Heldring, et al., 2014). Similar findings have been reported for proteomic analysis of ECM from aged rat striatum, with increases in Hapln1, brevican, and versican (Raghunathan et al., 2018, 2020). Levels of hyaluronan, a glycosaminoglycan that is a major component of the brain's ECM, also increase in the brain of aged rodents (Reed et al., 2018; Sugitani et al., 2021).

In contrast to the consistent reports of increased ECM proteins in aged brain, reports on the density of WFA labeling of CS (Hartig et al., 1992) vary by brain region. WFA labeling in aged rodents increases in striatum (Richard et al., 2018), and inferior colliculus (Mafi et al., 2020), but decreases in auditory cortex (Brewton et al., 2016; Cisneros‐Franco et al., 2018). Disparate findings have also been reported for hippocampal subfields, with increased WFA labeling in aged CA1 (Vegh, Heldring, et al., 2014), but decreased WFA labeling in aged CA3 (Gray, Zempare, et al., 2023). In addition to changes in CS density, aged mice exhibit changes in the sulfation pattern of CS, with a shift toward patterns that may inhibit neuronal growth and plasticity (Foscarin et al., 2017; S. Yang et al., 2021). These changes to CS may have important functional consequences, as the density and sulfation of CS side chains are major factors regulating CSPG function (Dyck & Karimi‐Abdolrezaee, 2015; Galtrey & Fawcett, 2007; Yu et al., 2018).

Importantly, these aging‐related changes to ECM may contribute to cognitive aging. Excess accumulation of ECM may contribute to cognitive impairment in aged rodents, as PNNs and ECM generally favor circuit stability and impair synaptic plasticity, which impairs learning and memory (Fawcett et al., 2019, 2022; Reichelt et al., 2019). Thus, ablation of ECM improves learning and memory in aged rodents or in mouse models of Alzheimer's disease (Richard et al., 2018; Vegh, Heldring, et al., 2014; Yang et al., 2015). However, accumulation of ECM may be protective against oxidative stress (Suttkus et al., 2014) and lipofuscin accumulation (Gray, Zempare, et al., 2023; Morawski et al., 2004), and some studies indicate a protective effect of PNNs on cognitive function in aged rats (Gray, Zempare, et al., 2023) and nonhuman primates (Gray, Khattab, et al., 2023). Changes to ECM sulfation may also contribute to aging‐related cognitive impairment. C6 sulfation of CS is associated with plasticity, and aging‐related loss of C6 sulfation drives impaired performance on memory tasks in aged mice (S. Yang et al., 2021).

In this study, we replicated prior reports of increased aggrecan immunoreactivity in several brain regions of aged mice (Figure 5, Figure S4), and observed an increase in aggrecan and related ECM proteins in EV fractions from the same mice. This may indicate an association of ECM‐enriched EVs from aged mice with aging‐related changes to the brain's ECM, with the caveat that we analyzed EVs from an entire hemibrain, while aging‐related changes to the brain's ECM vary by brain region. Future studies analyzing EVs from specific brain regions could further test this association.

While ECM proteins are generally thought to be secreted directly from glia and neurons in the brain (Fawcett et al., 2019; Tewari et al., 2022), prior studies have reported the presence of ECM proteins in some EV subtypes (Chun et al., 2021; Kowal et al., 2016). Though not well understood in the brain, EVs are involved in formation and remodeling of ECM elsewhere in the body (Lewin et al., 2020; Rilla et al., 2019). Matrix vesicles are a specialized type of EV (Shapiro et al., 2015) that are critical for bone calcification (Golub, 2009). Similarly, EVs may contribute to calcification of blood vessels in cardiovascular disease (Bakhshian Nik et al., 2017), and can promote vascular amyloidosis by interacting with ECM (Whitehead et al., 2023). EVs also promote cardiac fibrosis by stimulating collagen production in fibroblasts (Datta et al., 2017; Yang et al., 2019), and regulate extracellular matrix during wound healing in skin (Cabral et al., 2018). EVs can also influence ECM remodeling by delivering proteases to ECM in various tissues (Shimoda & Khokha, 2017).

Based on the known interaction of EVs and ECM in other parts of the body, it might be possible that ECM‐enriched EVs in aged mice contribute to the age‐related changes in the brain's ECM. More investigation will be needed to test this hypothesis, and to determine whether brain EVs may contribute to cognitive aging. Given the important role of ECM in aging‐related diseases such as cancer (Cox, 2021) and neurodegenerative diseases (Amontree et al., 2023; Sun et al., 2021), the possible contribution of ECM‐enriched EVs to aging‐related diseases is another topic for future study.

## AUTHOR CONTRIBUTIONS

Conceptualization: AEA, Investigation: AKK, KK, JAM, and AEA, Formal analysis: CFM, KK, JAM, and AEA, Writing–original draft preparation: AEA, Writing–review and editing: AKK, CFM, KK, JAM, and AEA.

## FUNDING INFORMATION

This work was supported by a pilot grant from UAB's Integrated Center for Aging Research, National Institute on Aging grants R00AG056597, P20AG068024, and P30AG086401, and National Cancer Institute grant P30CA013148.

## CONFLICT OF INTEREST STATEMENT

The authors declare no conflicts of interest.

## Supporting information


Appendix S1.

Table S1.

## Data Availability

Data from proteomic analysis of mouse brain EVs have been deposited with the ProteomeXchange Consortium via the PRIDE partner repository (Perez‐Riverol et al., 2022) with dataset identifiers PXD052797 and DOI 10.6019/PXD052797. Other data are available from the authors upon reasonable request.
