# Supplementary material for: Increased levels of extracellular matrix proteins associated with extracellular vesicles from brains of aged mice
Source: Aging Cell. 2024 Oct 8;24(1):e14359. doi: 10.1111/acel.14359 (PMC11709096; doi:10.1111/acel.14359)
Supplement: Supplementary file 1 — Appendix S1. [file ACEL-24-e14359-s001.docx]

**Supplementary Material for:**

**Increased levels of extracellular matrix proteins associated with extracellular vesicles from brains of aged mice.**

Azariah K. Kaplelach^1^, Charles F. Murchison^1^, Kyoko Kojima^2^, James A. Mobley^2^,^3^, and Andrew E. Arrant^1^

1 - Center for Neurodegeneration and Experimental Therapeutics, Alzheimer’s Disease Center, Evelyn F. McKnight Brain Institute, Department of Neurology, University of Alabama at Birmingham, Birmingham, AL, USA

2 – Institutional Research Core Program/Mass Spectrometry, University of Alabama at Birmingham, Birmingham, AL, USA

3 – Department of Anesthesiology and Perioperative Medicine, University of Alabama at Birmingham, Birmingham, AL, USA

**Figure S1**

**Figure S2**

**Figure S3**

**Figure S4**

**
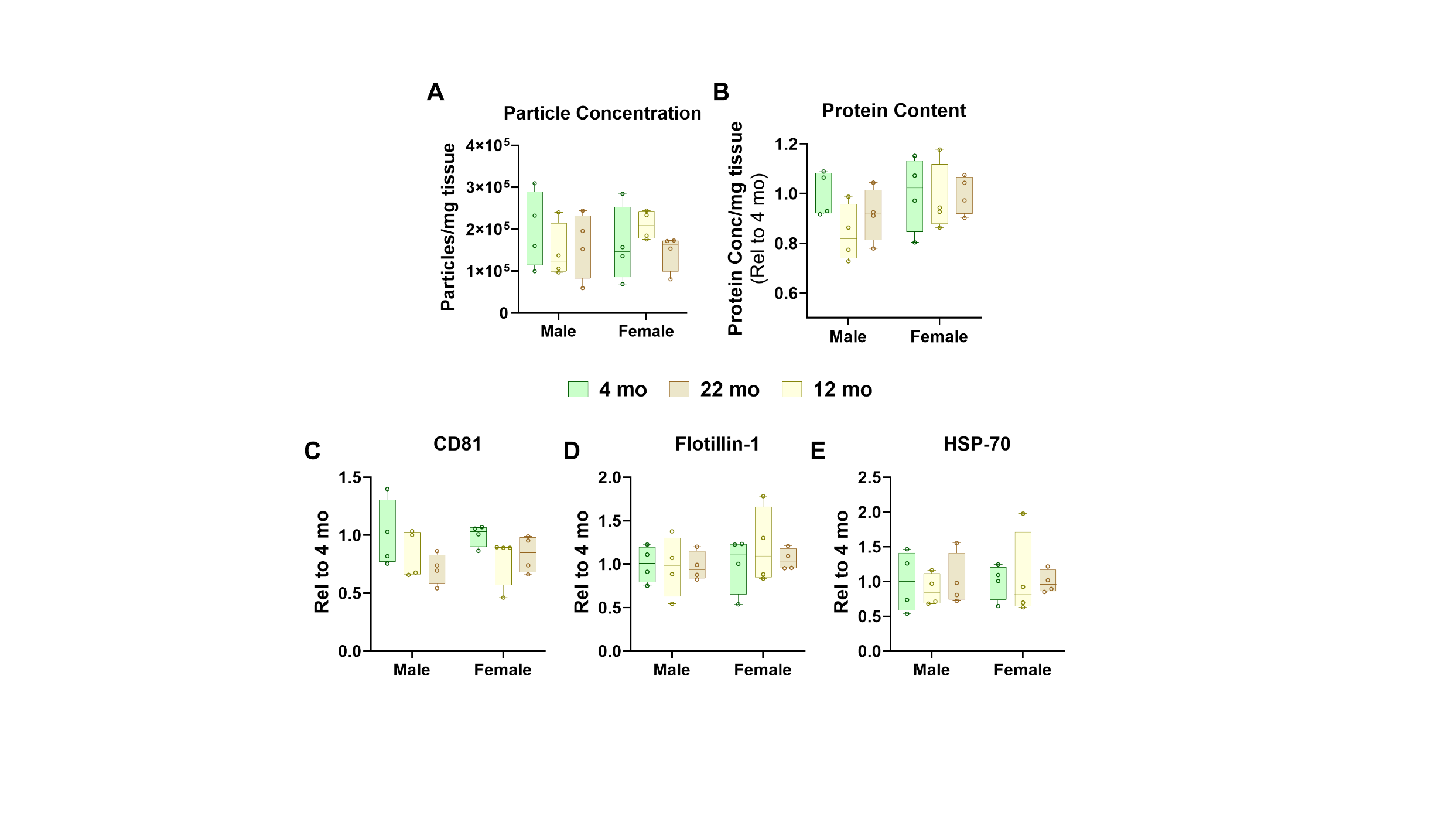
**

**Figure S1 – No sex differences detected in brain EV levels.** No significant age or sex differences were detected when the concentration of EV particles (**A**, ANOVA, effect of age, *p* = 0.7102, effect of sex, *p* = 0.933, age x sex interaction, *p* = 0.3245), EV total protein content (**B**, ANOVA, effect of age, *p* = 0.3035, effect of sex, *p* = 0.1324, age x sex interaction, *p* = 0.4913), or levels of the EV markers CD81 (**C**, ANOVA, effect of age, *p* = 0.0681, effect of sex, *p* = 0.7731, age x sex interaction, *p* = 0.6302), flotillin-1 (**D**, ANOVA, effect of age, *p* = 0.8224, effect of sex, *p* = 0.3959, age x sex interaction, *p* = 0.7284), or HSP-70 (**E**, ANOVA, effect of age, *p* = 0.9034, effect of sex, *p* = 0.7161, age x sex interaction, *p* = 0.9754) were analyzed with sex as a factor. n = 8 mice per age group (4 males and 4 females). Graphs present the data shown in Fig. 2 with male and female mice plotted separately.


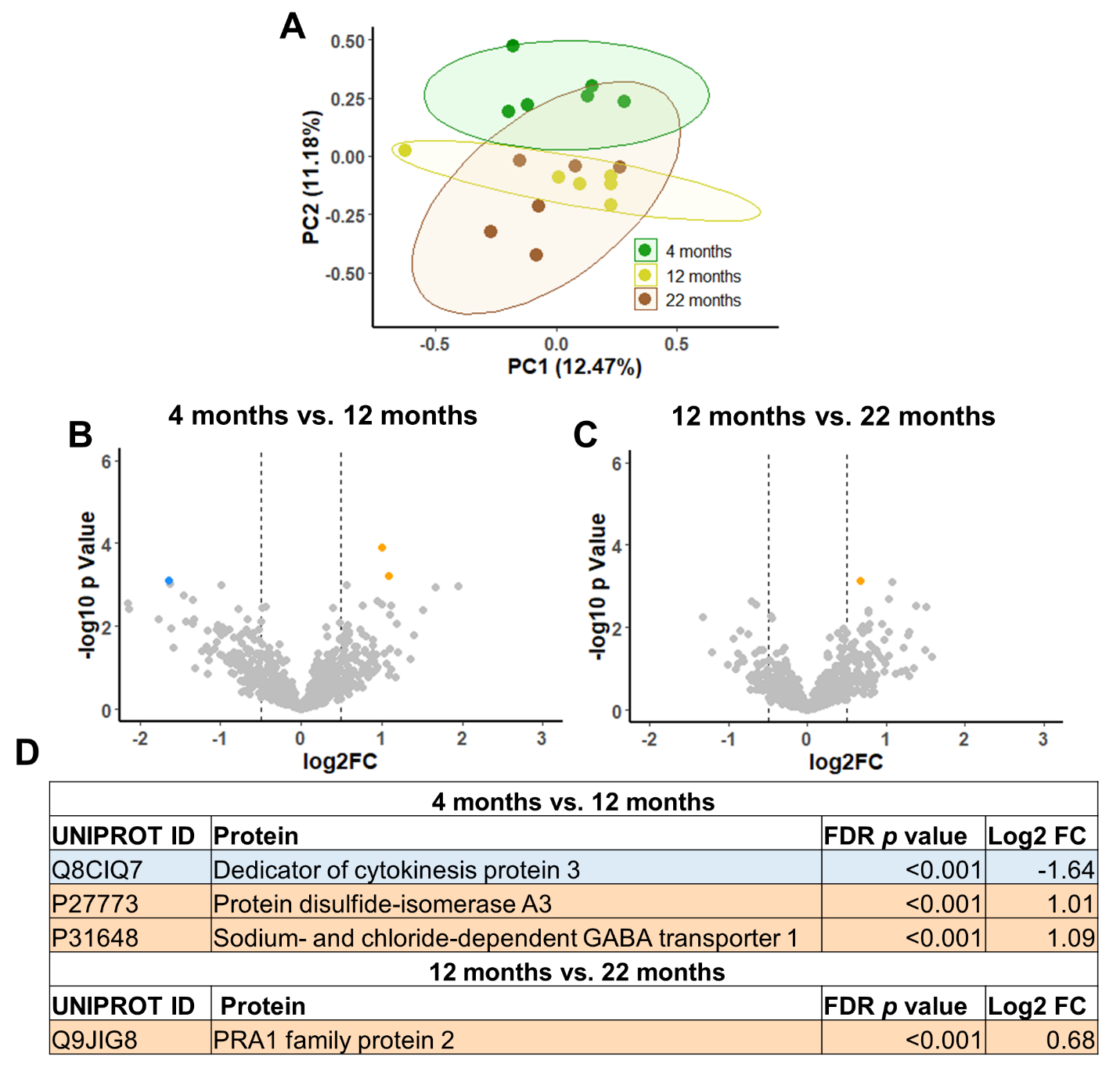


**Figure S2 – Pair-wise comparisons from EV proteomic analysis.** **A**, Principal components analysis revealed distinct clustering of EVs from 4-month–old mice and mice from older age groups. **B**, The EV fraction from brains of 12-month–old mice contained three proteins with differential abundance from the EV fraction from 4-month–old mice. **C**, The EV fraction from brains of 22-month–old mice contained only one protein with differential abundance than the EV fraction from 12-month–old mice. **D**, Summary of proteins with differential abundance from pair-wise comparisons in **B** and **C** (FDR-corrected *p* value < 0.1). Proteins increased in the older group are shaded orange and proteins decreased in the older group are shaded blue. Ellipses in **A** represent 95% confidence levels.


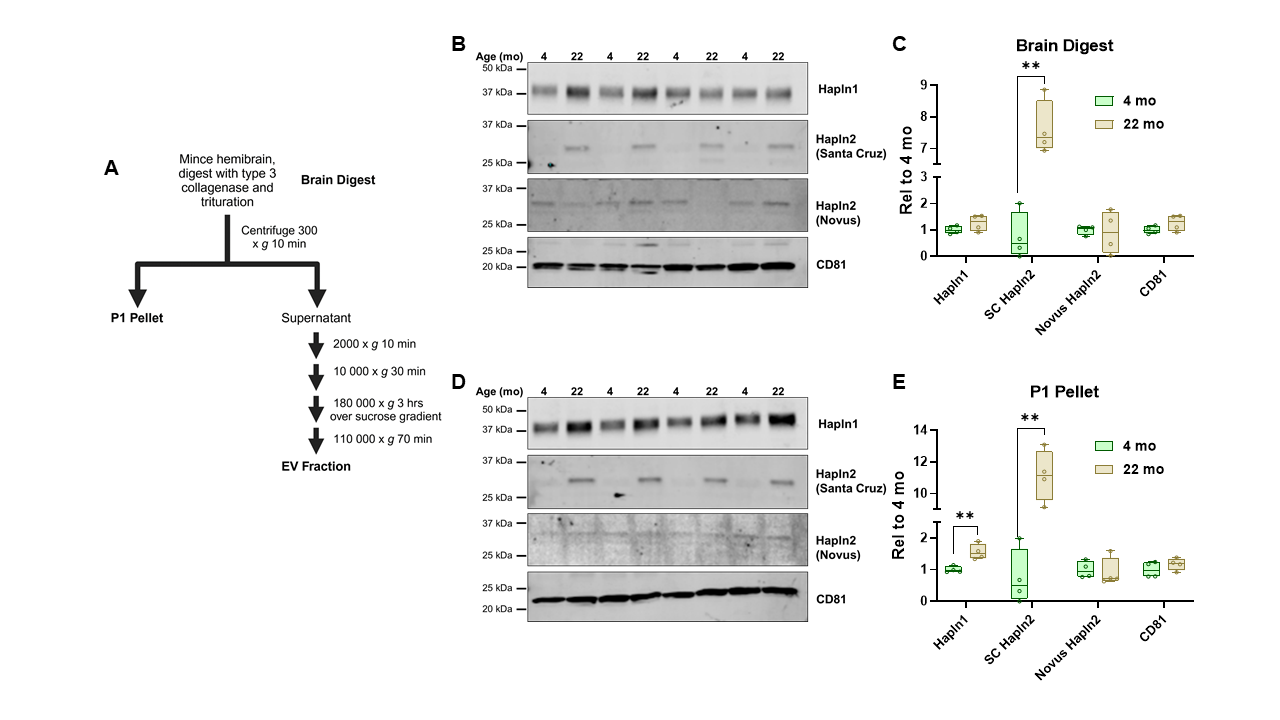


**Figure S3 – Levels of Hapln1 and Hapln2 in brain tissue.** **A**, Levels of Hapln1 and Hapln2 were assessed by immunoblot of the crude brain digest obtained as the first step of EV isolation and from the P1 pellet obtained from the initial 300 x *g* spin. We detected no age differences in Hapln1 levels of the brain digest (**B**, **C**, *t* test, *p* = 0.1848), but detected an increase in Hapln1 of the P1 pellet from 22-month–old mice (**D**, **E**, *t* test, *p* = 0.0031). Unlike in the EV fraction, the two antibodies used to detect Hapln2 produced different results. The Santa Cruz Hapln2 antibody detected almost no Hapln2 in 4-month–old mice, but a clear band in 22-month–old mice in both brain digest (**B**) and P1 pellet (**D**), resulting in a dramatic increase Hapln2 levels in 22-month–old mice in both fractions (**C**, brain digest, *t* test, *p* = 0.0036, **E**, P1 pellet, *t* test, *p* = 0.0019). In contrast, the Novus Hapln2 antibody detected faint bands in both fractions (**B**, **D**), with no age differences in either brain digest (**C**, *t* test, *p* = 0.4067) or P1 pellet (**E**, *t* test, *p* = 0.6067). Levels of CD81 did not differ between age groups in either fraction (**C**, brain digest, *t* test, *p* = 0.1848, **E**, P1 pellet, *t* test, *p* = 0. 2929). n = 4 mice per age group, 2 males and 2 females. Y axes on **C** and **E** are broken to allow clearer visualization of the distribution of data from all proteins. Panel **A** created with BioRender.com.


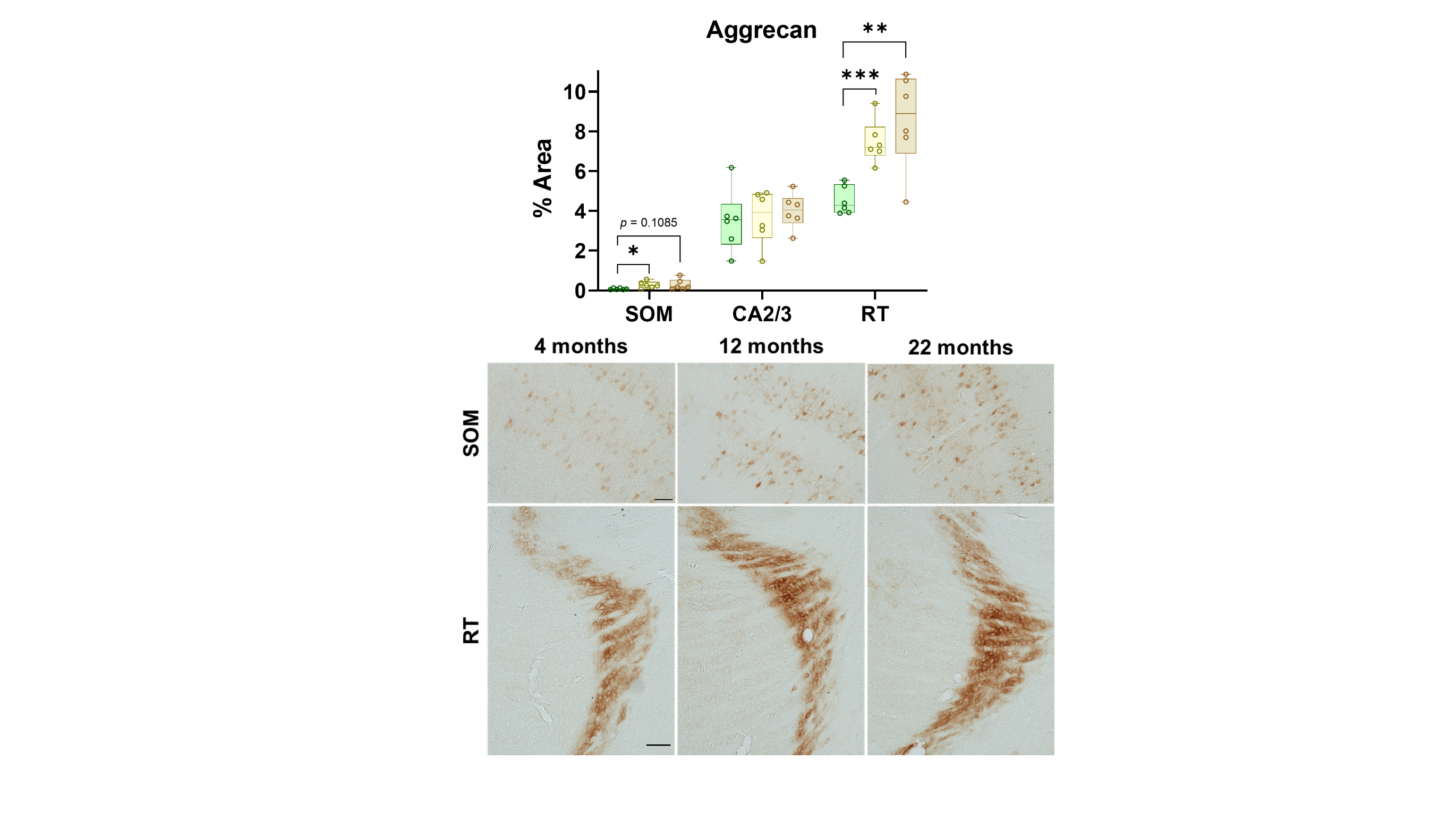


**Figure S4 – Chromogenic immunostaining for aggrecan.** Analysis of chromogenic immunostaining for aggrecan revealed an increase in the total area of staining with age (RM ANOVA effect of age, *p* = 0.0139). In somatosensory cortex, 12-month–old mice had a greater area of immunolabeling than 4-month–old mice (* = *p* = 0.0159 by Dunnett’s post-hoc test), with a similar trend in 22-month–old mice (*p* = 0.1085). Both 12- (*** = *p* = 0.0003 by Dunnett’s post-hoc test) and 22-month–old (** = *p* = 0.0086) mice had a greater area of aggrecan immunolabeling in the reticular nucleus of the thalamus than 4-month–old mice. n = 6 mice per age, 3 males and 3 females. Scale bars represent 100 μm.
